# The Review of Current and Proposed Methods of Manufacturing Fir Tree Slots of Turbine Aero Engine Discs

**DOI:** 10.3390/ma16145143

**Published:** 2023-07-21

**Authors:** Jarosław Buk, Paweł Sułkowicz, Dariusz Szeliga

**Affiliations:** 1 Faculty of Mechanical Engineering and Aeronautics, Department of Manufacturing Techniques and Automation, Rzeszow University of Technology, 35-959 Rzeszow, Poland; sulkowicz@prz.edu.pl; 2 Faculty of Mechanical Engineering and Aeronautics, Department of Materials Science, Rzeszow University of Technology, 35-959 Rzeszow, Poland; dszeliga@prz.edu.pl

**Keywords:** fir tree slot, broaching, CFG, WEDM, AWJ, ECDM, COVID-19, EDM, WECM, Wire ET

## Abstract

This review article presents a summary of currently used and proposed methods of manufacturing fir tree slots of discs in turbine engines. The production of aircraft, including aircraft engines during times of overlapping global economic crises related to the COVID-19 pandemic or the war in Eastern Europe requires a quick response to the changing numbers of passengers and cargo. Similarly, the aviation industry must adapt to these conditions, and thus utilize flexible production methods allowing for a quick change in the design or type of a given part. Due to the constant adoption of new materials for the most critical aero engine parts and the necessity of complying with environmental regulations, it is necessary to search for new methods of manufacturing these parts, including fir tree slots. As an alternative to currently used expensive and energy-intensive broaching, many manufacturers try to implement creep feed grinding CFG or contour milling. However, other manufacturing methods, thus far rarely used for crucial machine parts such as WEDM, ECDM or AWJ, are gaining more and more popularity in the aviation industry. This article presents the advantages and shortcomings of these methods in the context of manufacturing fir tree slots.

## 1. Introduction

The continuous growth in the number of passengers and cargo transported by plane is reflected by increased demand for new aircraft. As a result, air transport is one of the fastest-growing sources of greenhouse gas emissions. At the same time, more and more strict environmental regulations and standards are being introduced. Research into less polluting fuel is being conducted. New aircraft designs strive for reducing the size and weight of structures as well as introduce aerodynamic improvements, thus lowering the amount of fuel used. As a result, among others, sustainable aviation fuels (SAFs) have been introduced, allowing to reduce greenhouse gases by 80% compared to fossil jet fuel [1,2,3,4,5].

**Figure 1:**
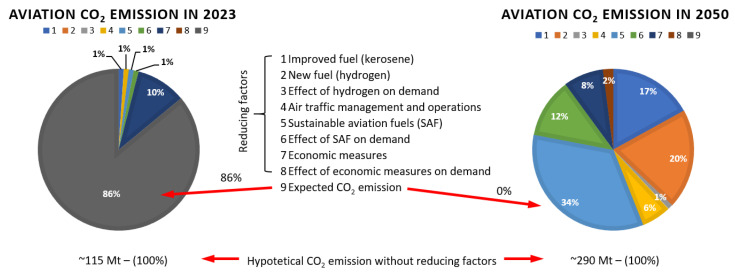
Expected areas of aero engine development in order to decarbonize air transport in Europe according to the Destination 2050 project—A route to net zero European aviation.

The Clean Aviation program of the European Union assumes the introduction of a hybrid electric drive in the smallest regional aircraft by 2035, which will ultimately allow a reduction in fuel consumption of 50% and CO_2_ and NO_x_ emissions of 90% for this type of aircraft. In addition, the use of ultra-efficient aircraft for short- and medium-distance travel is assumed, allowing low amounts of fuel to be consumed or even the use of low/zero-emission energy sources such as synthetic fuels or non-drop-in fuels such as hydrogen. One of the objectives of the project is to design the drives (including engines using these fuels—liquid hydrogen in particular), which, in turn, would reduce fuel consumption by 30% and CO_2_ and NO_x_ emissions by 86% for this type of aircraft, and ultimately reduce CO_2_ emissions to zero by 2050 (Figure 1). Similar changes concerning zero-emission flights are to be implemented by Airbus by 2035 [2,6,7,8,9,10].

**Figure 2:**
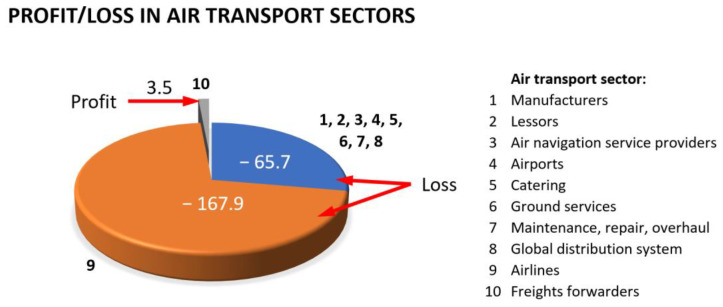
Economic profit/loss by subsector during the first year of the COVID-19 pandemic in USD billions.

Another factor to consider is variation in passenger and cargo transport resulting from unforeseen global events, such as pandemics or wars, which can make it unprofitable for a company to maintain a fleet of wide-body aircraft with a very large number of seats. In the case of the COVID-19 pandemic, air transport, at its peak, decreased by 60% [11]. Figure 2 presents the economic balance of individual industry sectors related to air transport during the first year of the COVID-19 pandemic [12].

The arguments stated above indicate the need to replace wide-body aircraft with smaller ones or to implement new solutions in the design of the engines and their efficiency, which would affect the profitability of their use even during such impactful events. These changes can already be seen as the production of the wide-body Boeing 747 (commonly referred to as a jumbo jet) has ceased as of 2022. Events such as pandemics or wars also increase the need to adopt more flexible production methods, allowing for quick responses adequate to the current needs of the supply chain [11,13].

**Figure 3:**
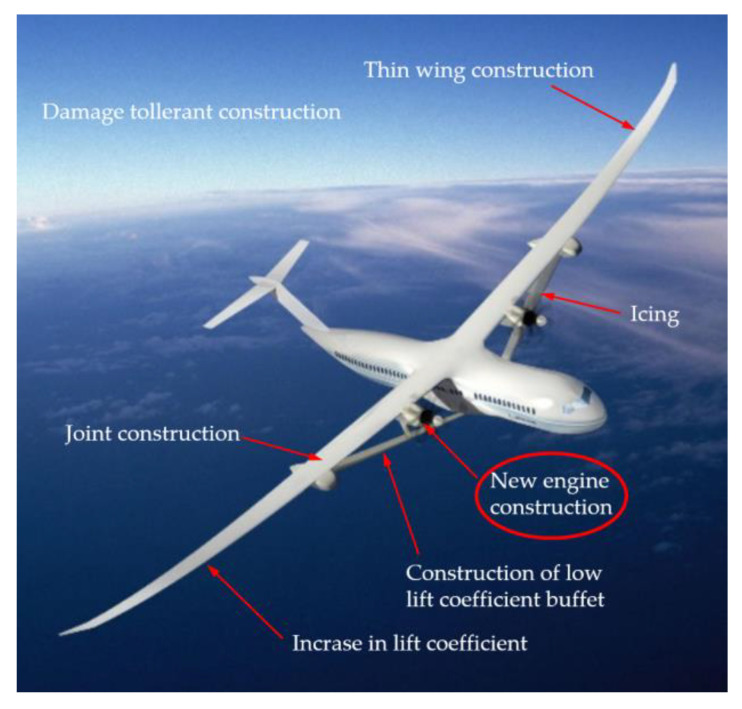
The Transonic truss-braced wing (TTBW) concept and the selected development challenges of the project.

At the same time, NASA, in cooperation with Boeing, announced the introduction of a new concept of short- and medium-range aircraft in the joint Sustainable Flight Demonstrator (SFD) project. The project involves a new generation hull concept design of a green, single-aisle airliner, namely the transonic truss-braced wing (TTBW) concept (Figure 3). It is a concept of an aircraft with extra-long, thin wings stabilized by diagonal struts.

The aim of such a design is to lower fuel consumption due to the shape of the wings and, consequently decrease drag. New solutions include the design of the engine as well, which will be located in the plume–strut interaction area. The prototype is set to be built in the second half of the 2020s and mass-produced in the 2030s [14,15].

One of the possible ways to reduce engine emissions is the development of hybrid electric engines. The combination of a jet engine and an electric motor on the one hand aims to reduce pollutant emissions and noise, and on the other hand, is supposed to reduce maintenance costs. The technology already developed by NASA, GE Aerospace and Boeing is compatible with sustainable aviation fuel. In 2022, the hybrid electric propulsion with hydrogen fuel cells project was presented [16].

**Figure 4:**
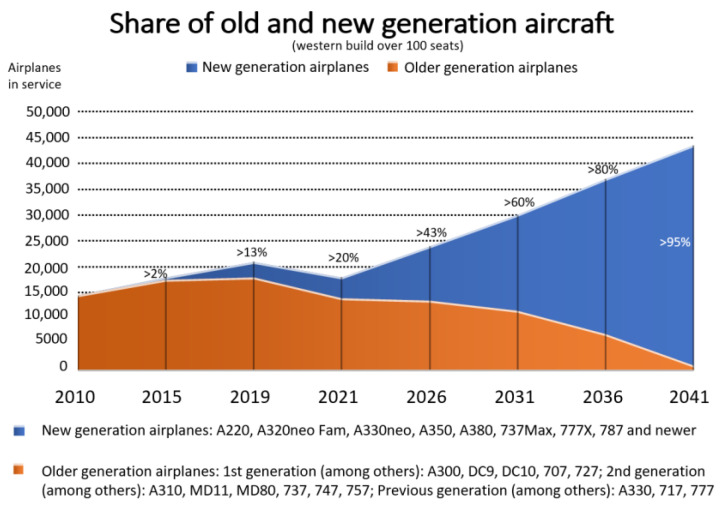
Estimated share of new-generation aircraft according to Airbus GMF.

Airbus, in the Global Market Forecast (GMF) for 2022–2041, predicts a 2-fold increase in air cargo over the next 20 years. The two largest aircraft manufacturers, Airbus in the GMF and Boeing in the Commercial Market Outlook (CMO) 2022–2041, predict that air passenger transport will return to the levels before the COVID-19 pandemic as early as 2024/2025 and will increase approximately 2-fold by 2041. As a result, about 40,000 new passenger and cargo aircraft would have to be manufactured during this time. Currently, new generation of aircraft account for only 20% of all aircraft in use. According to the predictions, in the following 20 years, the rate will change to >95% (Figure 4) [17,18].

**Figure 5:**
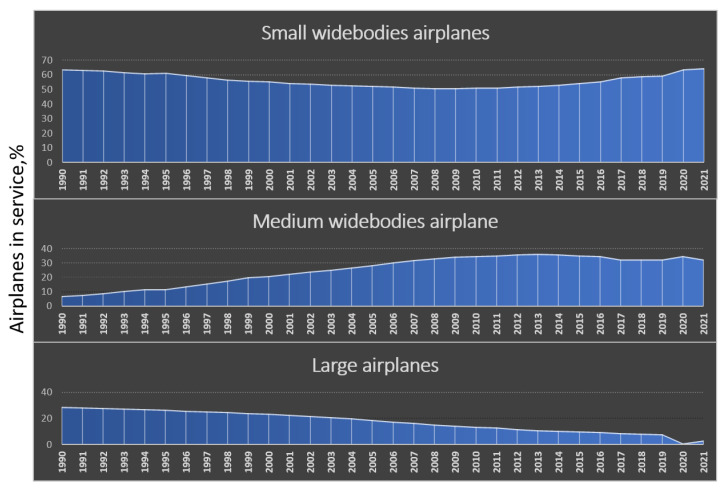
The share of the largest, medium and small aircraft among all wide-body aircraft according to Boeing CMO.

Boeing, in CMO 2022–2041 (Figure 5), indicates that the historical relationship between the number of passengers and aircraft size has changed. Previously, long routes and a large number of passengers required, for economic reasons, wide-body aircraft equipped with three or four engines. Currently, the trend is changing, and the developments in airframe and engine design allow for smaller, twin-engine, wide-body aircraft to be used even for the longest routes [18].

In addition, according to the International Air Transport Association (IATA), the average age of the global commercial jet fleet has increased from 6.3 years in 2000 to 11.8 years in 2021 and is estimated to increase to 15.1 years by 2035. As aircrafts age, they will require more maintenance and repair work, creating opportunities for suppliers of spare parts in the global aero engine market. Therefore, it is important to search for flexible methods of manufacturing these elements due to the small series of spare parts [19].

In order to fulfill those expectations, the aviation industry incorporates newer materials and structures in aircraft design. Aero engines and related components are one of the most critical parts of an aircraft. Thus, these materials are increasingly difficult to shape when machining. Therefore, the production of these parts proves to be challenging as well. The parts must be manufactured in accordance with the regulations and safety requirements of the aviation industry. The engine design has thus undergone an evolution from high-strength steel parts to titanium alloys or heat-resistant super alloys (HRSA) with a high nickel content such as Inconel. Companies manufacturing aero engine components allocate significant resources in the search for technologies that could improve and speed up the production of aircraft engines. Currently, one of the most recent development is the use of 3D printers for manufacturing, among others, ceramic fuel nozzles and fuel systems [11]. One of the goals of the European Union’s Large Passenger Aircraft Program is the introduction of new technologies focused on the integration of the latest most fuel-efficient propulsion concepts in aero engines and airframe structures aimed at short- and medium-range aircraft. New solutions include open rotor engine architecture, ultra-high bypass ratio (UHBR) turbofans and hybrid propulsion concepts [20].

Turbine discs are parts of the aero engine characterized by complex design and requiring special manufacturing tools. The blades are attached to the turbine disc with fir tree slots. Up to the end of the first decade of the 21st century, broaching has been the predominant method of manufacturing fir tree slots in turbine discs. Due to the costs and time necessary to implement the technology of machining a new fir tree slot geometry, research on alternative manufacturing techniques has been intensified in the following years. Although broaching continues to be the leading fir tree slot manufacturing method, other methods are slowly gaining their share in the overall production of turbine discs. In 2009, the Unconventional Advanced Manufacturing Processes for Gas-Engine Turbine Components (ADMAP-GAS) project was initiated, aimed at finding new manufacturing methods for fir tree slots. As a result, abrasive water jet cutting (AWJC) was proposed as a method of roughing machining and high-speed wire electrical discharge machining (HS-WEDM) for the finishing manufacturing of the slots [21].

**Figure 6:**
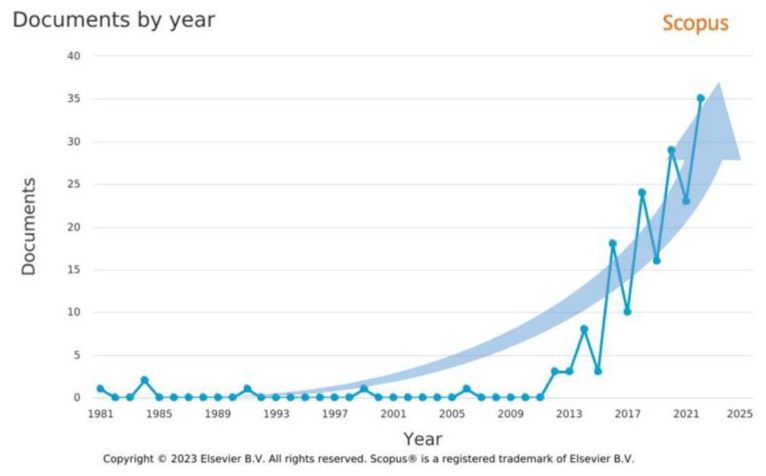
The number of publications in the Scopus core database regarding fir tree slots over the years 1981–2022.

Significant developments in the design of aero engines resulting from a large number of factors influencing the increasingly complex requirements imposed on the aviation industry contribute to the considerable increase in the interest in issues related to fir tree slots in recent years. Figure 6 presents a graph of the number of publications containing the phrase “fir tree slot” in the Scopus database between 1981 and 2022.

In conclusion, important factors directly influencing air transport, and indirectly affecting the design of both aircraft and engines, are predictable factors such as increasingly strict regulations regarding exhaust emissions and environmental protection. However, certain global events such as wars or pandemics have a significant impact as well. Some can cause a significant decrease in passenger or cargo transport, while others could increase it. For economic reasons, being able to react quickly to such changes is of great importance. Regarding the design of aircraft, or more specifically aero engines, the production of their parts using conventional methods suited for the previous era may become unprofitable and will require the use of new methods. In the case of the production of turbine blades, the interest in their production has increased several dozen times. Thus, the aim of this paper was to present the current and proposed methods of manufacturing fir tree slots in turbine discs. This objective arises from the need to replace the expensive broaching, which is currently the leading slot manufacturing method. Alternative methods show a lot of promise in terms of machining accuracy and surface quality at lower operating costs. In addition, they can be more adaptable in the area of utilized tools and the design of the manufacturing process.

## 2. Design of the Aero Engine Turbine Unit

**Figure 7:**
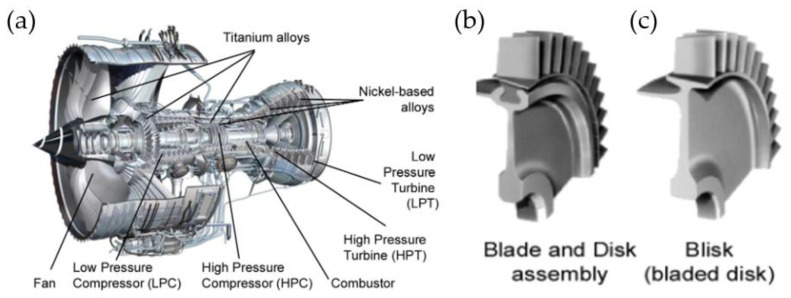
Section of a turbofan engine (**a**), assembled disc (**b**), bladed disc (**c**) [24] “Reprinted/adapted with permission from Ref. [24]. 2014, Elsevier”.

The turbine engine is used in transportation methods such as air, sea and land, as well as in the heating industry. The part that determines the engine’s parameters to the greatest extent is the gas turbine. A higher gas temperature reduces specific fuel consumption per unit of power. Thus, materials with increasingly high heat resistance and strength are needed and as a result, new manufacturing methods are needed as well [22]. One of the basic components of the aero engine are low- and high-pressure turbines. Each turbine consists of a series of bladed discs, which may be assembled or in the form of a monolithic bladed disc (Figure 7) [23].

**Figure 8:**
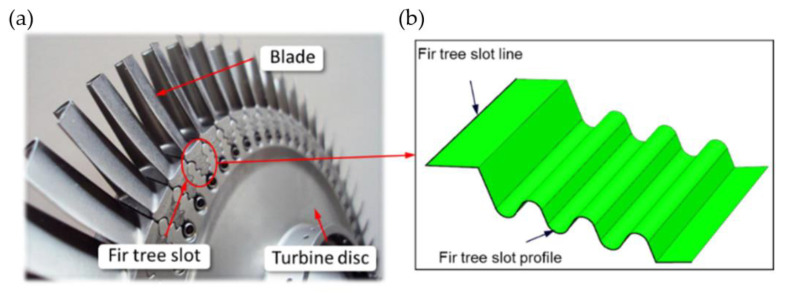
Design of the assembled disc (**a**), disc slot surface (**b**) [25].

An assembled disc consists of blades mounted in the disc with slots (Figure 8a). The type of slot depends on the operating conditions and location (turbine and compressor stage). In compressor discs, the most widespread type of connection between the blade and the disc is the trapezoidal one, commonly referred to as the dovetail joint. Most of the damage for this type of connection results from vibrations of blades penetrating into the joint, leading to the formation of fatigue cracks. In turbines, however, joints between the disc and blades are usually of a fir tree shape. Such connections are less prone to damage resulting from vibrations due to more even distribution of a load on the surface of the joint. The correct connection between the blade and the disc occurs when proper contact with the entire surface of the individual joint protrusions is ensured. Contact conditions between the disc and blade surfaces are established via elastic–plastic deformation of roughness peaks and the layers located below. For the same material of the disc slot and the blade, contact conditions are highly influenced by the quality of the surface layer: surface roughness, stresses in the surface layer or the amount of surface strengthening resulting from the adopted manufacturing method [22]. The surface of the fir tree slot can be described by two lines (Figure 8b). The first line corresponds to the slot profile and the second is referred to as the fir tree slot line.

**Figure 9:**
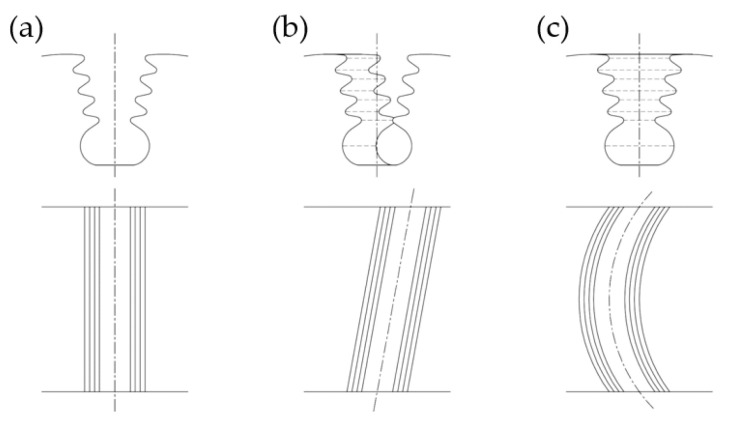
Turbine disc fir tree slot types: (**a**) straight, (**b**) diagonal, (**c**) arc.

The second line may be straight (parallel to the axis of the disc), diagonal (at an angle to the disc’s axis) or arc-shaped (Figure 9). Straight slots are used with slightly twisted blades whereas diagonal and arc-shaped ones are used with blades with strongly twisted bodies.

**Figure 10:**
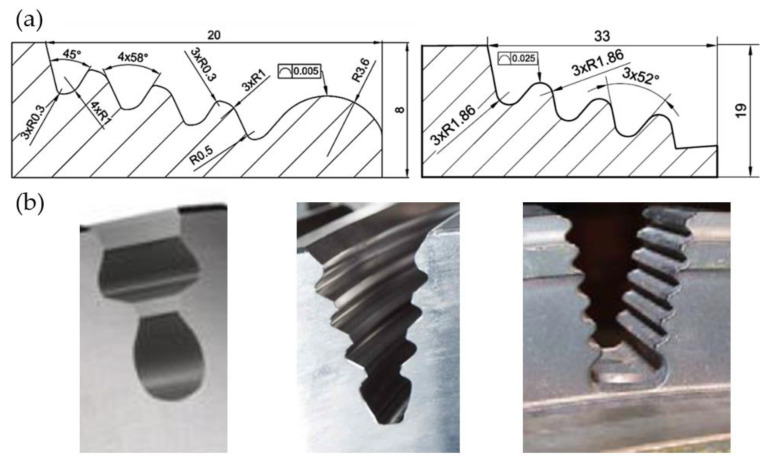
Examples of simplified technical drawings of slots (**a**) and profiles of turbine disc slots (**b**).

Narrow manufacturing tolerances and high surface quality are among the key features of fir tree slots. (Figure 10) [26]. The most commonly produced slots are of a width of 20 to 40 mm. The shape accuracy is in the range of 5 to 25 μm, and the surface roughness is in the range of 0.8 to 1.25 μm. The minimum inside radius is 0.3 mm. Usually, fir tree slots have two to seven pairs of protrusions, whereas in aviation applications, two or three pairs are the most common [27,28,29].

**Figure 11:**
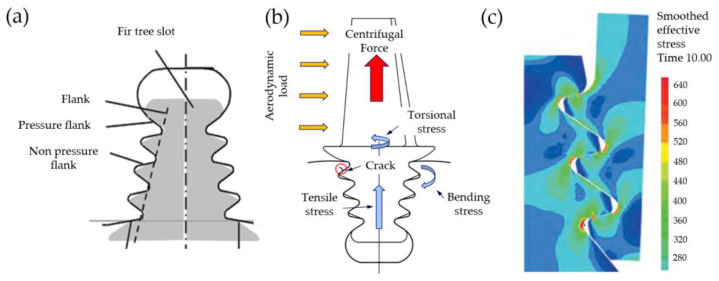
Fir tree slot and blade root assembly (**a**), turbine blade load (**b**), stress distribution in the blade root and disc slot (**c**) [33].

Currently produced engines can include from 2000 up to even 3500 blades. As a result, the reliability and durability of an engine depend mainly on the reliability of the blades and discs [27]. Fir tree roots and turbine discs are among the most heavily loaded parts of a turbofan engine. The most critical parts of the turbine are usually fir tree slots and the mounting holes of the turbine disc [30]. The blade is subjected to forces and loads, e.g., centrifugal force and aerodynamic load (Figure 11b), which induce significant operating stresses in the pressure flank area (Figure 11a). Gas dynamic loads characterized by time-dependent waveforms lead to an increase in vibrations and high-cycle fatigue [31,32]. These phenomena, in turn, lead to tensile, torsional and bending stresses (Figure 11c). In addition, high temperatures reduce tensile strength and yield strength. Adverse factors such as hot gases, combustion products and fuel additives, high pressure and temperature, centrifugal forces and vibrations lead to mechanisms that can destroy the slot, i.e., fatigue, abrasive and corrosive wear (fretting) or dust, water and gas erosion [33,34]. Therefore, no micro-cracks should be present in the surface layer of the slot. Moreover, it is recommended that compressive stresses occur in the surface layer. The design and manufacturing process of the fir tree slots has to be focused on stable operating conditions and the long service life of the turbine. For the reasons above, there are high demands concerning the properties, composition and quality of the materials used. One of the key features of these materials is a high strength, which to a large extent, makes them difficult to machine [22,27,35].

During the operating conditions of the turbine, the durability and reliability of the disc slots and blade roots depend, among others, on:

(a) Stress concentration;
(b) The materials used;
(c) The condition of the surface layer after machining, including surface roughness, surface layer stresses, amount of strengthening;
(d) The coating type and its properties.

Items (b)–(d) are directly related to the manufacturing process [27].

## 3. Methods of Manufacturing Fir Tree Slots of the Turbine Discs

Among the currently used methods of manufacturing fir tree slots in the turbine discs, machining leads the way, primarily broaching but milling and grinding as well. Electrochemical machining (ECM) can be distinguished among the less frequently adopted methods [22]. Due to high manufacturing costs, new methods are constantly being sought. The most promising new manufacturing methods in recent years are abrasive electrochemical machining (AECM), wire electrical discharge machining (WEDM) and abrasive water jet machining (AWJM) [21,24,36,37,38,39,40,41,42,43]. The requirements for fir tree disc slot manufacturing can be divided into two groups: requirements concerning machining accuracy and those regarding surface quality, such as surface roughness or the thickness of the white layer. Current technologies of producing fir tree slots require the use of etching and shot peening. Electrolytic etching is used to detect segregations in the surface layer, which can promote the formation of microcracks under dynamic loads. Shot peening is aimed at reducing the stresses in the surface layer or inducing residual compressive stress. Thus, hardness changes, which increases fatigue strength and reduces vibration wear as a result of fretting corrosion. The above-mentioned operations reduce the risk of stress and fatigue corrosion cracking and are required by current certification standards [44]. A significant number of factors influencing an aero engine part and seeking alternative fir tree slot manufacturing methods, which are characterized by many parameters, require tests to be conducted using standard and advanced research tools such as deep learning techniques or grey wolf optimization [45,46]. Due to the increasing use of alternative slot manufacturing methods, it has been proposed to divide the individual stages of machining into pre-slotting roughing, semi-finishing and finishing [38]. In order to achieve the necessary quality of turbine discs, their manufacturing process should ensure that obtaining parameters such as Ra ≤ 0.8 µm and cracks, discoloration, non-parent material is not permitted. Microstructural/metallurgical changes can be divided into three groups. The first comprises surface micro-anomalies. Contamination is allowed at a depth of less than 0.01 mm. Porosity is not allowed. The second group involves the heat-affected zone (HAZ). Recast layer, redissolution of phases, white layer, recrystallized zone and redeposit layer are not allowed in this zone. The last group comprises mechanical properties. In this case, abnormal residual stresses should assume values below σ ≤ 850 Mpa [38].

### 3.1. Broaching

**Figure 12:**
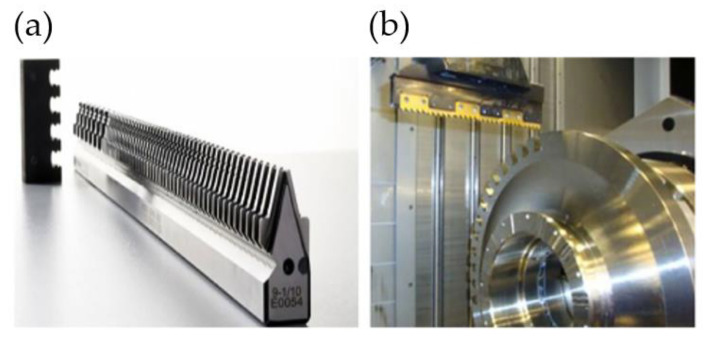
Broach: (**a**) broaching fir tree slots in turbine discs with a CNC machine tool (**b**) [43].

Currently, the most commonly used method of machining turbine fir tree slots is broaching. It is a process, during which the allowance for roughing and finishing is removed in a single machining pass of the broach. The tool can be situated horizontally or vertically, depending on the machine tool system [47,48]. The broach is usually made from high-speed steel (HSS), less often from carbide due to its brittleness [40,49,50]. Figure 12 presents an HSS broach and a view of the machining zone of a broaching machine tool.

**Figure 13:**
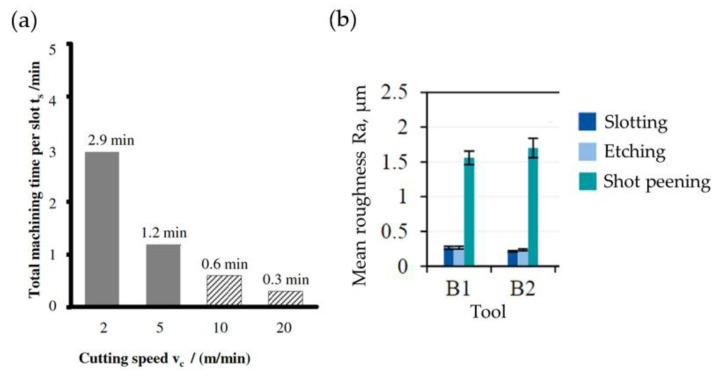
Pre-slotting time for HSS and cemented carbide broach—slot length 45 mm (**a**), surface roughness Ra after broaching with the new broach (B1) and with the used one (B2) (**b**) [40,44]. “Reprinted/adapted with permission from Refs. [40,44]. 2014, 2022, Elsevier”.

Vogtel et al. compared the machining efficiency of HSS and carbide broaches. Figure 13a presents the machining time of a single slot for both types of tools [49]. Küpper et al. studied the influence of carbide tool wear on surface roughness. Figure 13b shows the surface roughness Ra of Inconel 718 DA alloy after broaching with a new cemented carbide tool (flank wear VB = 0 μm) and with a used one (VB = 200 μm). In order to unify the surface layer and reduce stresses, etching and shot peening were conducted. Etching did not influence surface roughness Ra in a significant way, but shot peening resulted in an over 6-fold increase in Ra compared to only broaching.

**Figure 14:**
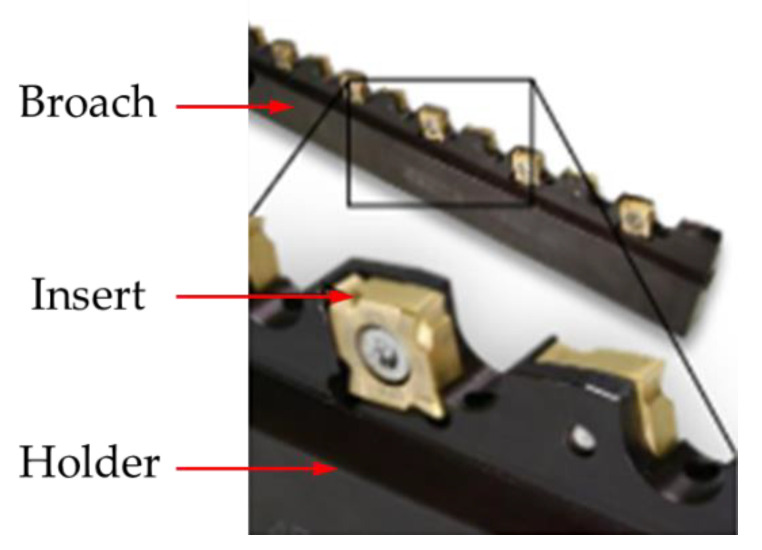
Broach with indexable cemented carbide cutting inserts [48]. “Reprinted/adapted with permission from Ref. [48]. 2020, Elsevier”.

The advantages of broaching include high machining accuracy, surface quality, machining efficiency and repeatability. However, broaching is a crucial process in the production chain of turbine discs. Significant cut-layer cross-sections generate very high cutting forces, reaching up to 10,000 N, which in turn causes rapid tool wear and high energy consumption. To achieve proper machining conditions, broaches are often sharpened and new protective coatings are applied [51,52,53,54]. Considering the above, Klocke et al. proposed a tool with indexable cemented carbide cutting inserts (Figure 14) [55].

**Figure 15:**
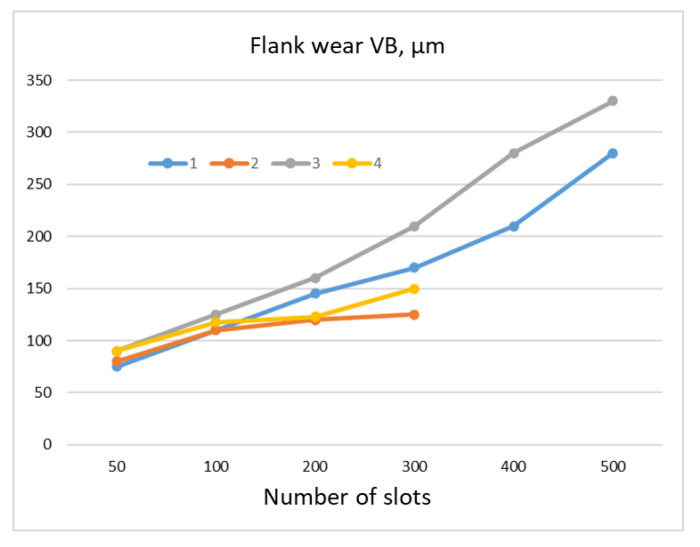
Cemented carbide cutting insert wear depending on the grade and cutting speed: 1—grade 420, *v_c_* = 18 m/min, 2—grade 360, *v_c_* = 18 m/min (chipping after 300 slots), 3—grade 420, *v_c_* = 12 m/min, 4—grade 360, *v_c_* = 12 m/min (chipping after 300 slots).

Figure 15 presents the wear of cutting inserts produced from different carbide grades: grade 420 and 360. A sample made from Allvac 718 plus alloy was machined. The slot width (machining length) was equal to 26.4 mm. Inserts made from 420-grade carbide allowed to twice as many slots to be machined, regardless of the cutting speed, in comparison with the 360-grade inserts [55].

**Figure 16:**
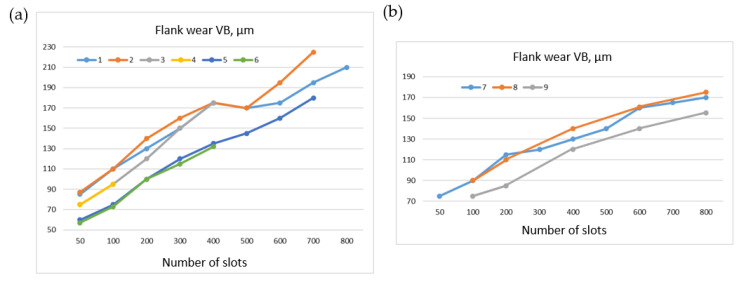
Wear of coated and uncoated cutting inserts when machining: (**a**) Allvac 718 plus: 1—TiN, *v_c_* = 18 m/min, 2—TiN, *v_c_* = 24 m/min, 3—TiN, *v_c_* = 30 m/min, 4—TiN, *v_c_* = 36 m/min, 5—uncoated, *v_c_* = 24 m/min, 6—uncoated, *v_c_* = 30 m/min, (**b**) Inconel 718: 7—TiN, *v_c_* = 24 m/min,8—TiN, *v_c_* = 30 m/min, 9—uncoated, *v_c_* = 24 m/min.

Figure 16 shows a comparison of the wear rate between cutting inserts with PVD coating from TiN and no coating during the machining of Allvac 718 plus and Inconel 718 alloys. The wear of uncoated inserts was faster. It was possible to machine more slots in the Inconel 718 sample at similar wear rates in comparison with the Allvac 718 plus sample. Inconel 718, therefore, was characterized by better machinability [55].

The use of a carbide broach increased the productivity approximately 2.5 times compared to HSS broach. The production cost decreased by almost half [56]. The above-described method is expensive due to costly machine tools and broaches. The design and manufacturing of a tool equipped with several thousand cutting edges for roughing, semi-finishing and finishing can take up to 9 to 12 months [39]. The low production flexibility of this method results from the necessity of making a new tool with each modification of the slot profile. All these factors determine the use of broaching primarily in high-volume mass production [22,28,38,57].

### 3.2. Milling

**Figure 17:**
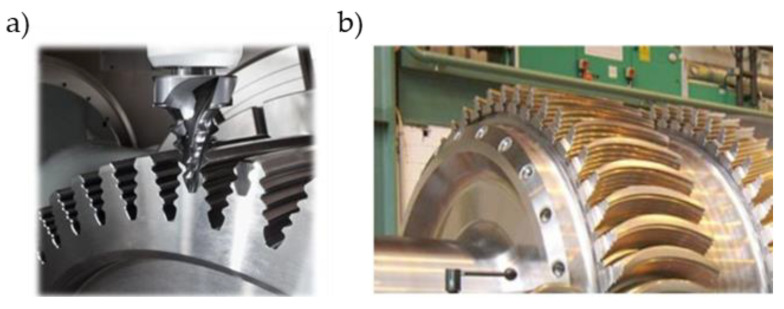
Milling fir tree slots in turbine discs: (**a**) straight shape, (**b**) arc shape [43].

The high costs associated with broaching have led to search for alternative methods of fir tree slots machining. One of them is trochoidal milling. The final slot profile is milled with a form-milling cutter (Figure 17). Cutting speed can reach up to 25–30 m/min with feed rate of 0.015 mm/blade. The advantage of this method is the possibility of machining arc fir tree slots [58,59,60,61,62].

**Figure 18:**
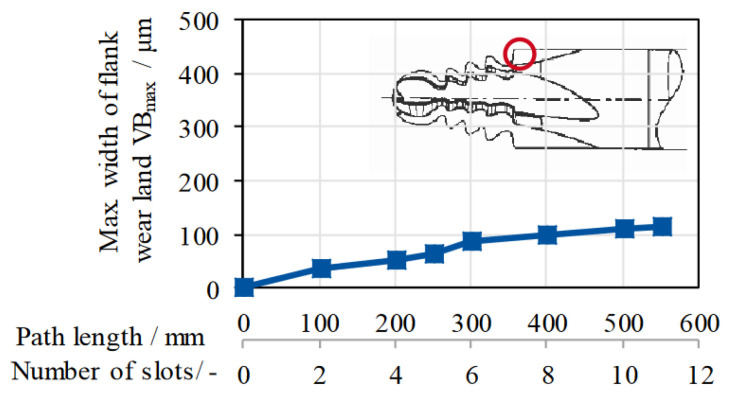
Wear of the carbide cutter in relation to path length/ number of slots [43].

Jianhua et al. noticed that milling in a single machining pass may lead to subjecting the cutter to very high thermal loads and, as a result, accelerated tool wear (Figure 18). Therefore, dividing the profile of the slot into a few parts and machining the subsequent sides and bottom of the slot was proposed [63,64,65,66]. Surface roughness after milling depending on the machining strategy is usually in the range of approximately Rz 2.1 μm to 5.9 μm [67,68,69].

**Figure 19:**
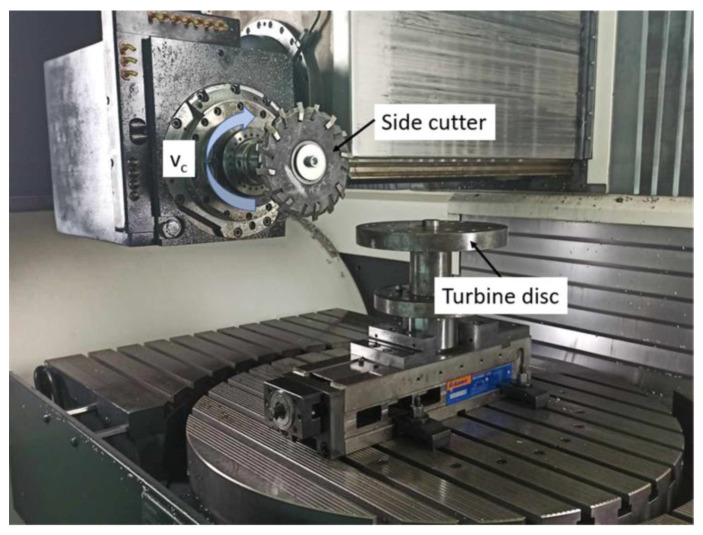
Pre-slotting with a side cutter with indexable ceramic cutting inserts.

Hence, Klocke et al. proposed the use of additional milling cutters to remove a significant amount of machining allowance in roughing machining (pre-slotting). The use of ceramic tools has been proposed as well, although their production is expensive. During machining, liquid cooling is not advised due to the risk of thermal shock, which could damage the tool. Only air cooling is used in order to remove cutting chips and prevent the formation of a built-up edge (BUE). Figure 19 presents the machining zone during pre-slotting [55,70].

**Figure 20:**
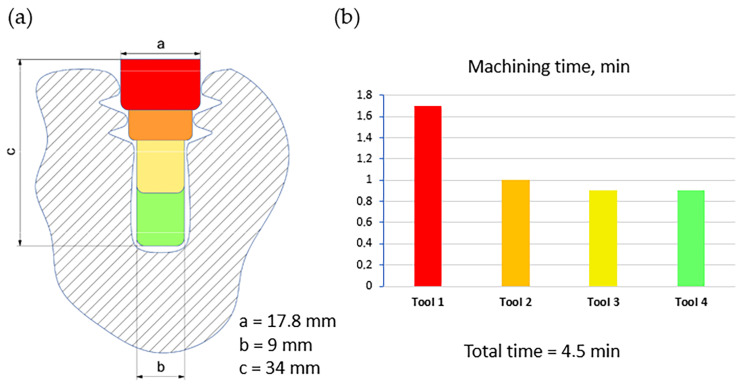
Initial trochoidal machining of slots—pre-slotting in milling: (**a**) milling trochoid strategy, (**b**) machining times in milling trochoid.

Figure 20 shows the milling trochoid strategy of material removal proposed by Klocke et al. and the machining time in comparison with the previously discussed broaching with HSS and carbide tools [39].

Machining is performed in four stages. The allowance is roughly removed with end mills and then a final profile of the slot is shaped with the form milling cutter [22,40]. This method allows tools that are easier to design and manufacture to be used, which increases production flexibility. However, the tool life is much shorter. Thus, machining a single slot this way is approximately 10 times more expensive than using broaching. Therefore, milling is used primarily in small-lot production [39,71].

### 3.3. Grinding

**Figure 21:**
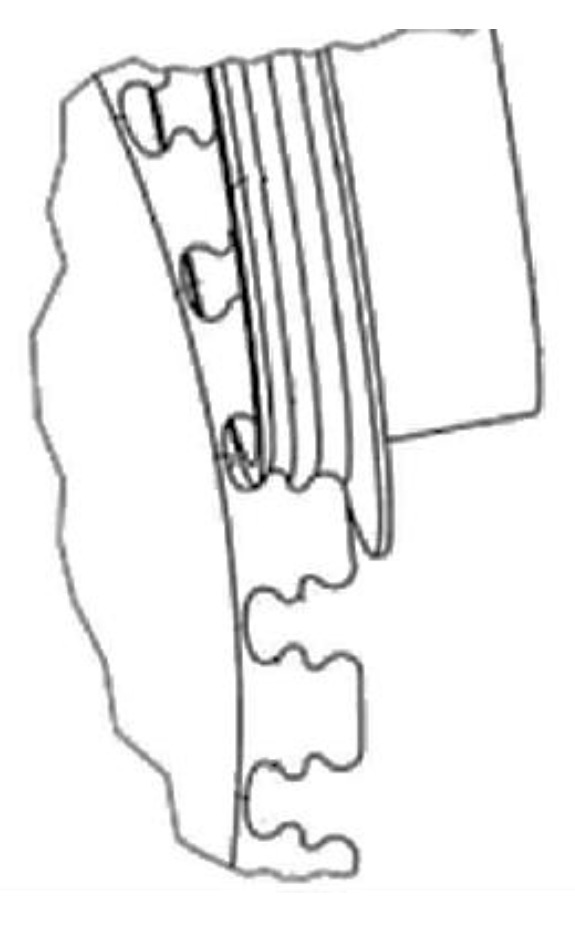
Grinding process with cup wheel [36]. “Reprinted/adapted with permission from Ref. [36]. 2009, Elsevier”.

Another fir-tree-slot-machining method is grinding. It can be divided into grinding with cup-grinding wheels or pin-grinding wheels. Grinding with cup wheels (Figure 21) applies only to the finishing machining of large-sized slots [36,72].

**Figure 22:**
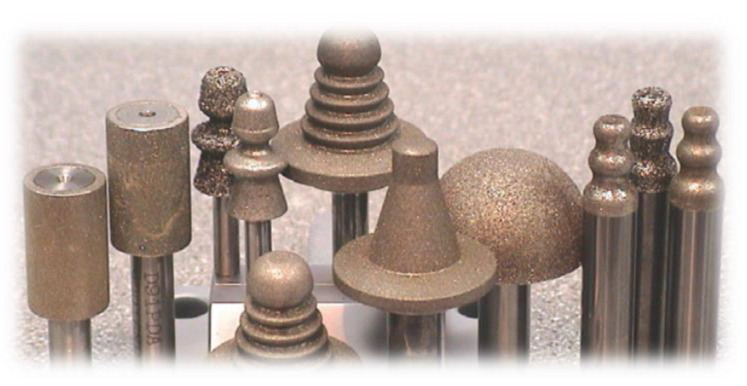
Pin grinding wheels with single layer diamond coating [76].

A more comprehensive solution was proposed by Aspinwall et al. It involves grinding with pin wheels with diamond (D46) and cBN (B46, B76, B91) coatings (Figure 22) [73,74,75]. Machining is divided into three stages. First, roughing takes place, with cylindrical wheels requiring the use of high-efficiency methods, such as creep-feed grinding (CFG) or high-efficiency deep grinding (HEDG). Then, form grinding with conical wheels is conducted. In the end, finishing machining with form grinding wheels of a shape corresponding to the selected slot profile is performed [76,77,78,79,80]. In some cases, instead of roughing and form grinding, wire electrical discharge machining (WEDM) can be used for initial slot machining. Small dimensions of grinding wheels require the use of high-speed spindles, usually above 50,000 rpm, in order to achieve the required peripheral speeds of the wheel. Feed rate can reach about 100 mm/min. Machining is conducted with high-pressure coolant flow supplied into the grinding zone. This machining method is sometimes referred to as VIPER (very impressive performance extreme removal) [51,81,82,83].

**Figure 23:**
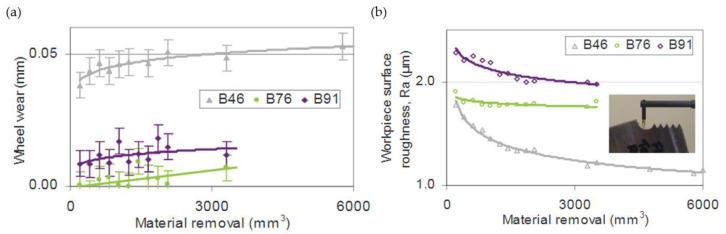
cBN grinding wheels’ wear (**a**) and surface roughness Ra after machining (**b**) [51]. “Reprinted/adapted with permission from Ref. [51]. 2007, Elsevier”.

In their research, Aspinwall et al. found out that the lowest wear was obtained for cBN grinding wheels—approximately 0.01 mm for B76 and B91. However, the lowest surface roughness has been registered for the B46 cBN wheel and it was equal to approximately Ra = 1.1 μm (Figure 23).

**Figure 24:**
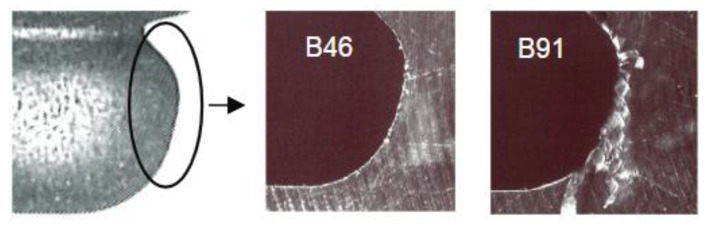
Entry burrs at 90,000 rpm for cBN B46 (no burr) and B91 grinding wheels [51]. “Reprinted/adapted with permission from Ref. [51]. 2007, Elsevier”.

The main disadvantage of this method involves rapid tool wear. Similar to milling, grinding a single fir tree slot is approximately 10 times more expensive than using broaching [39]. In addition, the risk of grinding burn, as well as entry burrs is high (Figure 24) [84].

**Figure 25:**
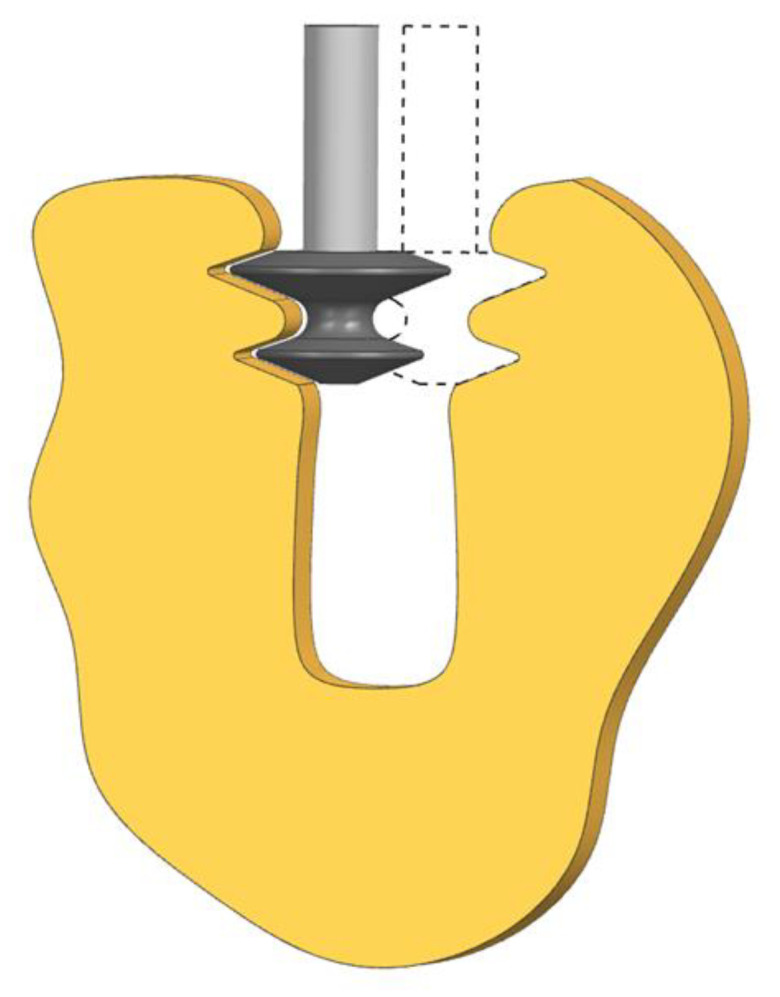
Single-sided, local-profiled grinding and electroplated cBN wheel.

Moreover, grinding with a profile wheel can be a part of a hybrid combination of different manufacturing methods, such as roughing WEDM, AWJ or EDM and finishing grinding. One solution is the proposed by Li et al. method consisting of roughing WEDM and finishing grinding using an electroplated cBN wheel [85]. The single-sided, local-profiled grinding method is presented in Figure 25. Due to the risk of burns, the grinding wheel with a diamond coating of a given grit size is responsible for the machining of only a part of the slot profile. Therefore, the entire finishing process requires the use of a set of grinding wheels.

**Figure 26:**
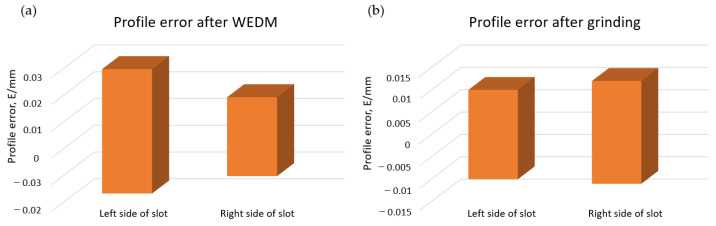
Profile errors after: (**a**) WEDM (**b**) single-sided, local-profiled grinding.

Figure 26 presents the shape deviations of the slot made of FGH69 superalloy after roughing WEDM (a) and finishing single-sided, local-profiled grinding (b). Accuracy was improved by more than 50%.

**Figure 27:**
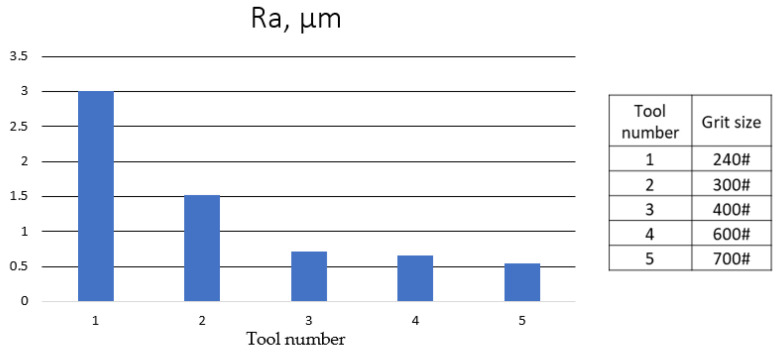
Surface roughness Ra depending on grit sizes of the electroplated cBN wheel [85].

The surface roughness largely depends on the diamond-grinding wheel’s grit size. The higher the grit size, the lower the surface roughness Ra (Figure 27). Acceptable surface roughness was achieved for grit sizes of 400 and above.

**Figure 28:**
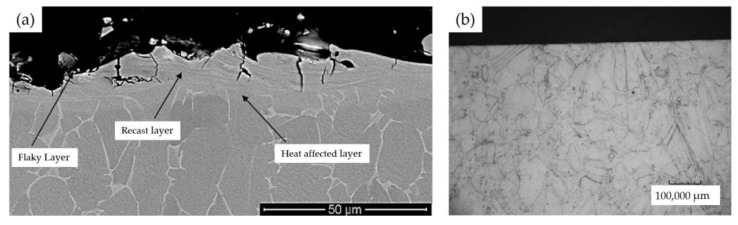
Microstructure: (**a**) after WEDM; (**b**) after grinding with cBN grinding wheel [86,87]. “Reprinted/adapted with permission from Refs. [86,87]. 2018, 2019, Elsevier”.

Figure 28 shows microstructures of samples after WEDM (Figure 28a) and after grinding with cBN grinding wheel (Figure 28b). A recast layer can often be observed after WEDM, whereas no such layer can be discerned after grinding.

### 3.4. Electrochemical Point Grinding

**Figure 29:**
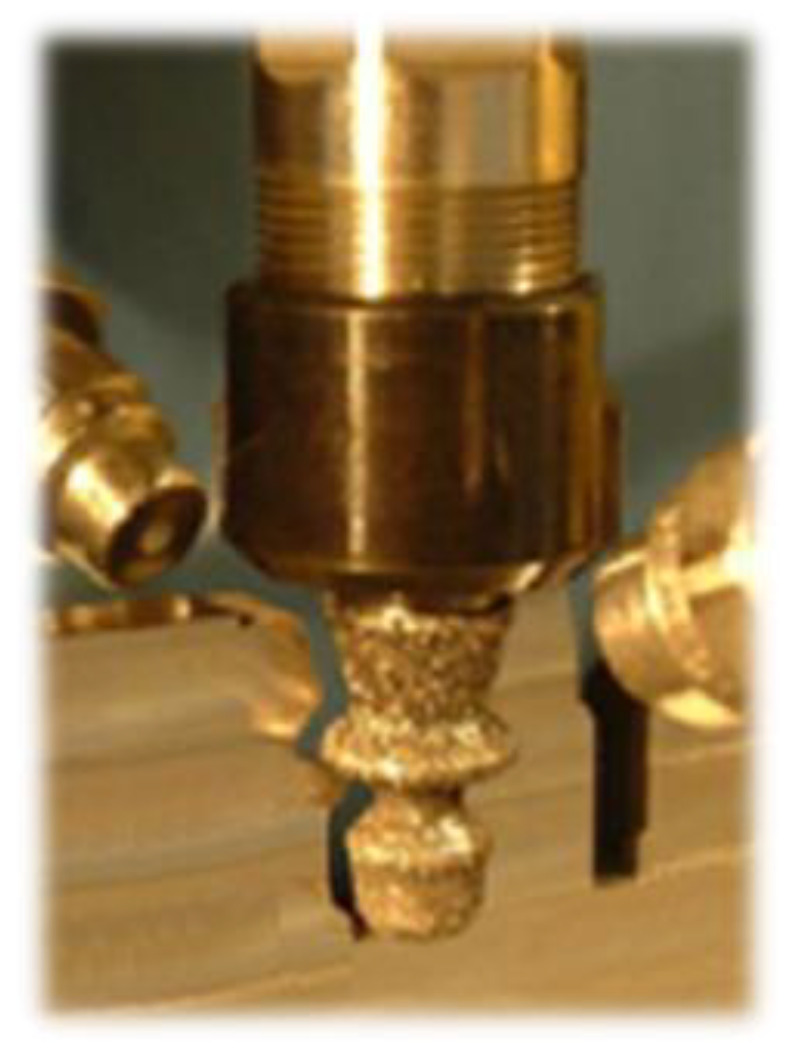
Pin-grinding wheel with cBN coating and visible nozzles supplying the electrolyte to the machining zone [37]. “Reprinted/adapted with permission from Ref. [37]. 2014, Elsevier”.

Electrochemical/electrolytic point grinding (ECPG) can be utilized for the finishing machining of fir tree slots. Depending on the slot profile and accuracy obtained after roughing machining, the finishing operation can involve electrochemical grinding with a form-grinding wheel (Figure 29). During machining, the slot is flushed with electrolyte from two nozzles. Using electroplated cBN grinding wheels (B151) with peripheral speeds of 11–15 m/s and an average grinding depth of 0.92 mm, when machining heat-resistant nickel alloys, surface roughness of Ra = 0.65 μm can be achieved [24,36,37].

**Figure 30:**
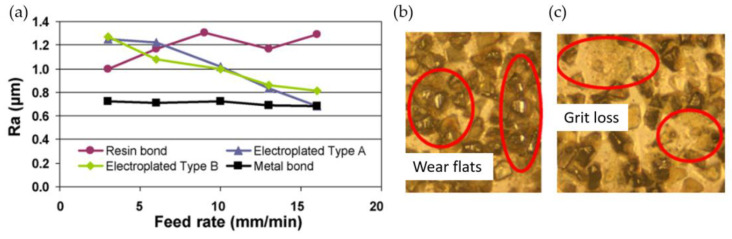
Surface roughness Ra after ECPG using grinding wheels with different bonds (**a**). Grinding wheel wear: (**b**) diamond coating; (**c**) cBN coating [36,88]. “Reprinted/adapted with permission from Refs. [36,88]. 2009, 2015, Elsevier”.

Ruszaj et al. and Curtis et al. describe the research of many teams on ECPG with difficult-to-cut materials. Figure 30a presents the surface roughness Ra of Udimet 720 ground using wheels with different bonds. The lowest roughness values were registered for wheels with metal bonds. Grit loss from cBN wheels is one of the key issues of the presented machining method (Figure 30c) [36,37].

**Figure 31:**
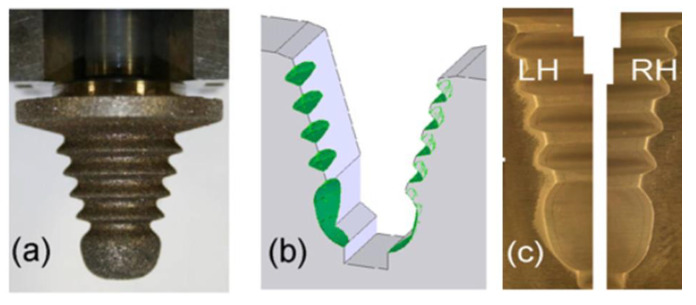
Electroplated cBN fir-tree-grinding point (**a**), CAD model of the contact zone at the entrance of the grinding wheel (**b**), left and right side of the entrance of the slot with visible oxides (**c**) [36]. “Reprinted/adapted with permission from Ref. [36]. 2009, Elsevier”.

In addition, the formation of oxides on the machined surface can occur (Figure 31), corresponding to electrolyte entry into the cut, which negatively impacts the mechanical properties of the slot.

### 3.5. Electrochemical Machining

**Figure 32:**
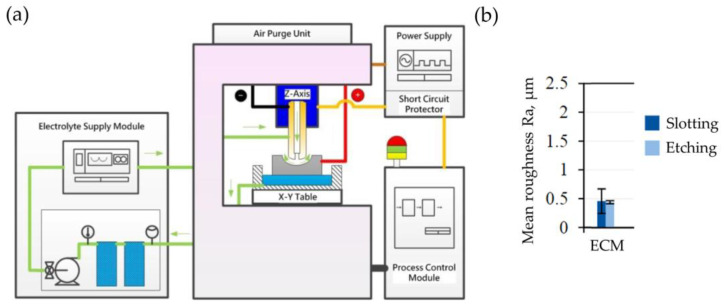
Scheme of the ECM (**a**); surface roughness Ra after ECM (*U* = 25 V, *v_f_* = 25 mm/min, *κ* = 159.4 mS/cm) (**b**) [44,89]. “Reprinted/adapted with permission from Ref. [44]. 2022, Elsevier”.

Electrochemical machining (ECM) can be adopted for roughing machining of slots (particularly in large-sized discs) due to its ability to machine any conductive material, regardless of its hardness. Other advantages include high machining efficiency, low tool costs and long tool life. Figure 32a presents the idea of electrochemical machining.

The material-removal mechanism is based on anodic dissolution during electrolysis. The process takes place in an electrolyte (e.g., NaCl solution), which is used to rinse the working gap. Figure 32b shows the surface roughness Ra after ECM of Inconel 718 DA alloy. After machining, etching was applied. It did not significantly influence the surface roughness Ra. Shot peening was omitted due to the lack of its impact on the mechanical properties of the workpiece [44]. When machining a fir tree slot with area of 252.17 mm^2^ and length of 40.89 mm, feed rate reaches from 0.25 to 2.25 mm/min, with effective material-removal rate of 2.34 mm^3^/(Amin) [90]. ECM allows a high-quality surface layer with no heat-affected zone, microcracks, changes in microhardness or internal stresses in the surface layer to be obtained. The main disadvantage of the process is the low machining accuracy. Therefore, it is implemented mainly as roughing machining before broaching, which allows the necessary number of broaches to be significantly reduced. One of the advantages of the ECM is the low cost of the tools [22,24,91].

### 3.6. Electrical Discharge Machining

**Figure 33:**
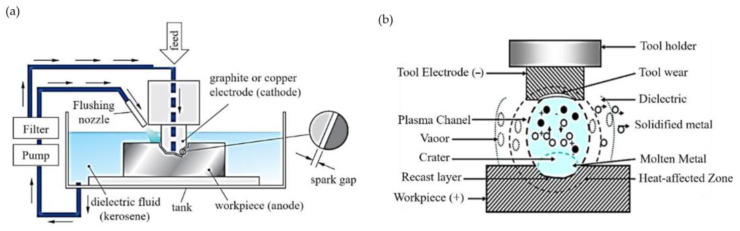
Electrical discharge machining: (**a**) diagram, (**b**) discharge model [94].

Electrical discharge machining (EDM) allows electrically conductive materials, with a conductivity greater than 0.01 S/cm regardless of their hardness and machinability, to be machined. The whole process takes place in a dielectric liquid, which is usually kerosene. Shaping the workpiece consists of removing a significant volume of material [91,92,93]. Figure 33 presents the EDM diagram and the discharge model.

Machining can be divided into roughing, finishing and superfinishing depending on the machining efficiency and the required condition of the surface layer [95,96,97]. The process can be characterized by a large number of parameters. Among the most important are discharge time, voltage and current, polarization of electrodes, size and rinsing conditions of the working gap [94,98]. When machining Inconel 718, surface roughness of approximately Ra = 8.5 um with material removal rate of 18.61 mm^3^/min can be obtained (In electrical discharge machining (EDM) of Inconel 718 by using copper electrode at higher peak current and pulse duration). The use of electrical discharge machining allows a surface layer with increased hardness in relation to the core material to be obtained, which can positively affect the operating conditions of turbine discs. However, high discharge energy determines the possibility of the occurrence of microcracks caused by tensile stresses in the surface layer, which may lead to accelerated fatigue wear. In addition, a white layer can appear in the surface layer, which is formed as a result of the solidification of the previously melted material [99,100,101]. For these reasons, the aviation industry does not allow this method for finishing machining of fir tree slots. It is also difficult to achieve the required surface roughness. Thus, EDM remains recommended for roughing machining of fir tree slots of turbine discs [22,57].

### 3.7. Wire Electrical Discharge Machining

**Figure 34:**
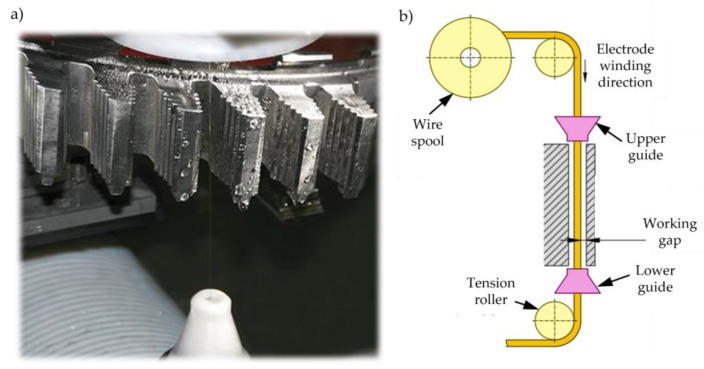
Wire electrical discharge machining of turbine disc slots (**a**), WEDM machine tool scheme (**b**) [43,119].

Wire electrical discharge machining (WEDM) is a type of electrical discharge machining, in which the tool is an electrode in the form of a wire, usually brass, with a small diameter of 0.02 to 0.5 mm (Figure 34). During operation, the wire electrode is constantly rewound and fed from the upper guide to the lower guide. As a result, there is always a new tool in the machining zone. The machining is conducted when the workpiece is immersed in a dielectric fluid, usually distilled water. Moreover, the dielectric is supplied in the form of a stream along the electrode from both guides in order to cool and isolate the electrode and to rinse the working gap from erosion products. As an alternative, a novel flushing mechanism has been proposed in recent years, where the dielectric is supplied to the machining zone as a stream [102,103,104]. The machining process consists of material removal as a result of melting and evaporation due to electrical discharges between the tool and the workpiece [105,106,107]. WEDM is a trepan-type technology, in which removing a significant amount of blank volume requires the erosion of a small amount of material [93,108]. In comparison with EDM, much lower discharge energy is needed in wire electrical discharge machining. Depending on the accuracy and surface roughness, the machining can be divided into rough, finishing and superfinishing (surfacing) cuts. Such an approach results in a lower heat impact, reduction of stresses in the surface layer and thus, a reduction in the micro-cracks [57,92,109,110]. The latest solutions in the design of the electrical discharge machine tools allow surface roughness Ra of 0.2 μm and HAZ thickness of nearly 0 μm [28,111,112] to be achieved. Inexpensive tools are another advantage of WEDM, allowing a single fir tree slot even cheaper than broaching, to be manufactured. The use of versatile tools and numerically controlled machine tools increases production flexibility due to the possibility of programing a new contour very quickly. Machining time, however, remains the main disadvantage of WEDM. Therefore, it is expected to be adopted in single and small-lot production [38,113]. Currently, wire electrical discharge machining is entering new industry sectors that were previously inaccessible. It is a direct result of improvements and developments of the technology over the last two decades in terms of surface quality and machining efficiency. For all those reasons, the use of WEDM for turbine disc manufacturing has been and is still the subject of research [24,41,114,115,116,117,118].

**Figure 35:**
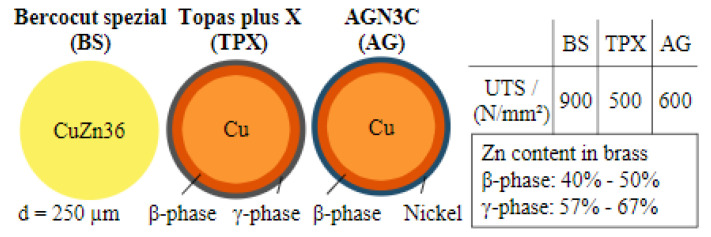
Electrode types used in the machining of Inconel alloys [28]. “Reprinted/adapted with permission from Ref. [28]. 2014, Elsevier”.

Due to turbine discs usually being made from Inconel alloys, of which the main element is nickel, Klocke et al. proposed the use of a coated electrode with an outermost layer made of nickel. The main advantage of this solution is the formation of a recast layer containing the material from both the workpiece and nickel electrode, instead of a mixture of nickel from the workpiece and copper or zinc impurities from the wire. Figure 35 presents a cross-section of the electrodes: typical brass Berocut spezial (BS), copper Topas plus X (TPX) with β-phase and γ-phase coatings and copper AGN3C (AG) with β-phase and nickel coatings.

**Figure 36:**
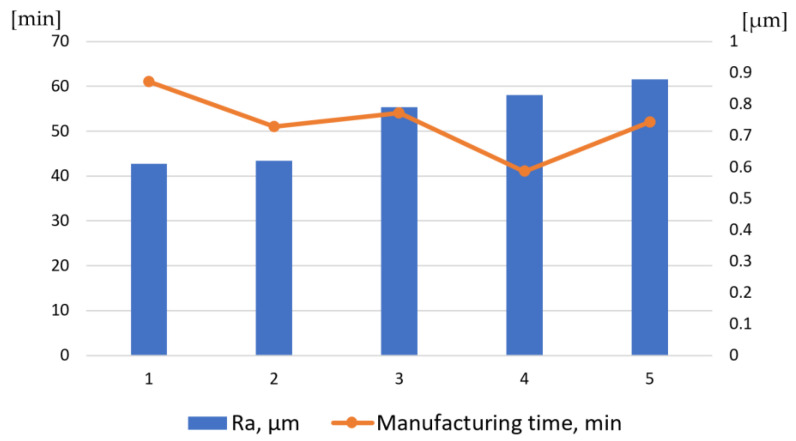
Surface roughness Ra and machining time with electrodes: BS (1—standard technology, 2—adapted technology), TPX (3—standard technology, 4—adapted technology), AG (5—adapted technology).

Figure 36 shows surface roughness in relation to the machining time of a single slot with BS (samples 1 and 2) and TPX (samples 3 and 4) electrodes, in standard and adapted technology. The results for the AG electrode are included as well (sample 5). The lowest surface roughness was achieved for the BS electrode and was equal to approximately Ra = 0.6 μm. The roughness of other samples was slightly higher—about Ra = 0.8 μm.

In WEDM, the height of the workpiece has the greatest influence on cutting speed, and therefore, on productivity. For a height of 5 mm (electrode diameter of 0.25 mm) the feed rate is about 6.4 mm/min when roughing. For finishing (four trim cuts) the feed rate varies from 4.6 mm/min to 6.1 mm/min. However, for a workpiece height of 30 mm, the feed rate drops to about 2.5 mm/min when roughing and in finishing (four trim cuts as well), it rages from 3.3 mm/min to 4.8 mm/min. Thus, a 25 mm change in height leads to a decrease in machining efficiency of about 60%. Comparing WEDM with HSS broaching, efficiency is understood as making the same slot is about 10% of broaching efficiency. The cost is about 75% of the broaching cost. However, production flexibility is about 8 times higher than with broaching [56].

**Figure 37:**
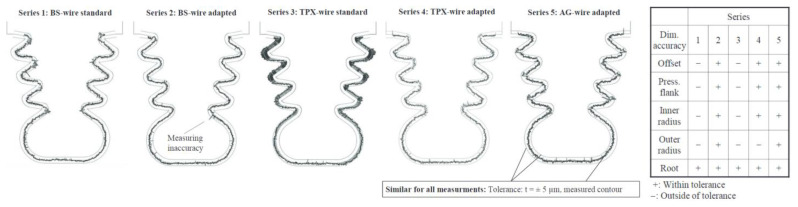
Distribution of deviations of samples made with electrodes BS BS (1—standard technology, 2—adapted technology), TPX (3—standard technology, 4—adapted technology), AG (5—adapted technology) [28]. “Reprinted/adapted with permission from Ref. [28]. 2014, Elsevier”.

The accuracy of machining (with the same electrodes) within a tolerance of t = ± 5 µm characterized tests 2 (BS adapted) and 5 (AG adapted). Figure 37 presents the distribution of deviations of the fir tree slot profile for all the samples.

**Figure 38:**
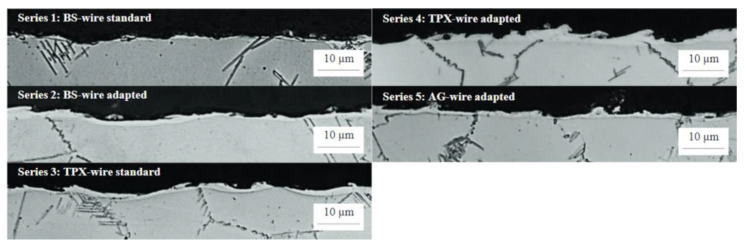
Thickness of the recast layer of samples made with electrodes BS BS (1—standard technology, 2—adapted technology), TPX (3—standard technology, 4—adapted technology), AG (5—adapted technology) [28]. “Reprinted/adapted with permission from Ref. [28]. 2014, Elsevier”.

Figure 38 shows a characteristic recast layer occurring in electrical discharge machining. The lowest thickness of the recast layer (below 1 μm) was achieved for sample 1 (BS standard). For samples 2 (BS adapted), 3 (TPX standard) and 5 (AG adapted) a slightly thicker recast layer was obtained (1–2 μm). For sample 4 (TPX adapted), the thickest recast layer was obtained (up to over 3 μm). A sample with no recast layer is very difficult to produce, especially in the inner radii of the slot profile [43].

**Figure 39:**
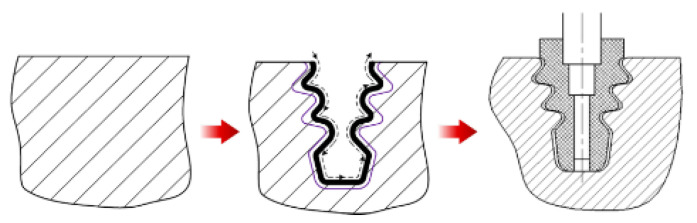
Hybrid technology combining WEDM roughing and finishing form grinding with a profile grinding wheel of a full slot shape [43].

Until the WEDM process overcomes the issues of the heat-affected zone (HAZ), it will be utilized mainly for roughing machining. Jianhua et al. proposed using form grinding as a finishing process after electrical discharge machining (Figure 39) [43].

### 3.8. Wire Electrochemical Machining

**Figure 40:**
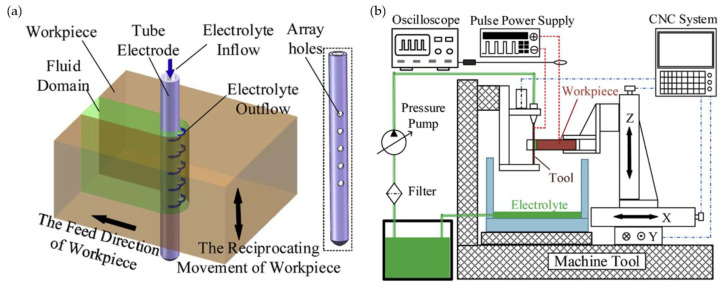
WECM scheme (**b**) using rotating helical electrode (**a**) [121]. “Reprinted/adapted with permission from Ref. [121]. 2019, Elsevier”.

A variation of ECM that can be utilized for machining fir tree slots with a wire electrode has been proposed by Jia et al. and Yang et al. The method is known as wire electrochemical machining (WECM). The process can be described as removal of core material (similar to WEDM) as a result of anodic electrochemical dissolution. The advantages of this method include no residual stress, the ability to machine materials regardless of their mechanical properties, very low or no tool wear, no heat-affected zone (HAZ) or recast layer [120]. Figure 40 presents a scheme of WECM.

**Figure 41:**
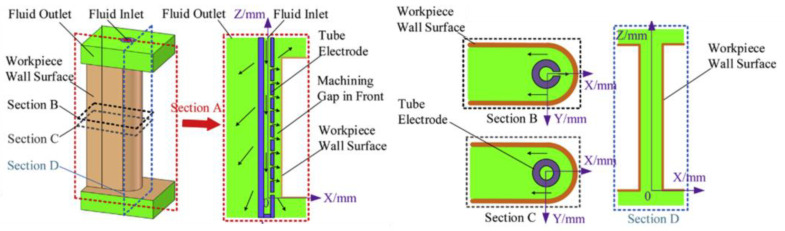
Electrolyte flow model in the machining zone [121]. “Reprinted/adapted with permission from Ref. [121]. 2019, Elsevier”.

Machining accuracy, surface quality and machining efficiency depend mainly on electrolyte, working gap flushing, electrical parameters, tool rotational speed, workpiece height and feed rate. The tool is usually made from tungsten due to chemical resistance and tensile strength [120,122]. A central hole supplying the electrolyte to the set of side holes (in the direction of the removed material), through which it enters the machining zone, is a characteristic feature of the process (Figure 41). The process is characterized by high feed rates of approximately 3.5 mm/min and material removal rate of about 882.6 mm^3^/min [43].

**Figure 42:**
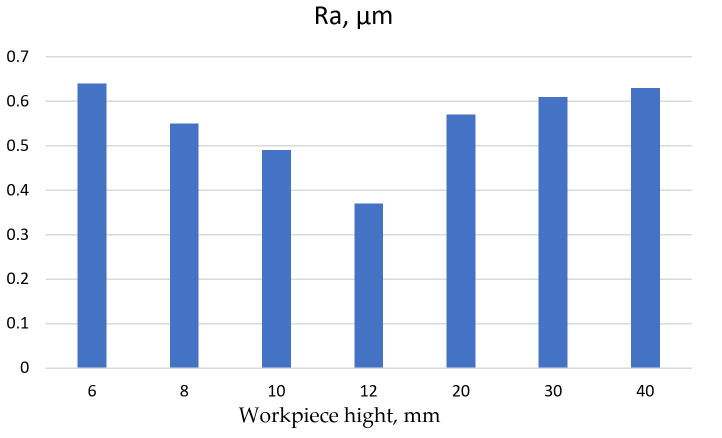
Surface roughness Ra depending on the height of the workpiece after WECM.

Surface roughness Ra obtained by Klocke et al. in their studies on WECM of Inconel 718 is shown in Figure 42 [123].

Regarding fir tree slots with a height of 40 mm, a satisfactory surface roughness Ra (below 0.8 μm) was obtained. In addition, this process has a negligible impact on the formation of HAZ [124].

### 3.9. Wire Electrochemical Trimming

**Figure 43:**
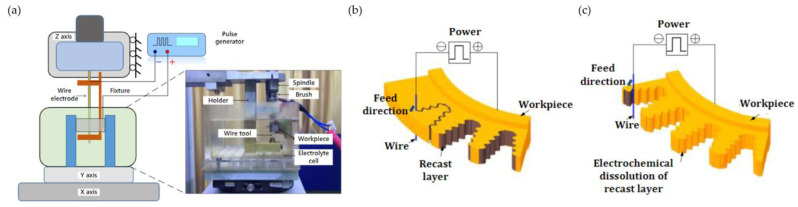
Wire ET scheme (**a**), scheme of the machining technology of fir tree slots consisting of WEDM pre-slotting (**b**) and Wire ET finishing (**c**) [126]. “Reprinted/adapted with permission from Ref. [121]. 2020, Elsevier”.

Considering the shortcomings of WEDM, mainly the formation of HAZ and recast layer, a hybrid slot machining method has been proposed [125]. Fang et al. conducted pre-slotting using WEDM and then removed HAZ and recast layer using wire electrochemical trimming (Wire ET) in several roughing, semi-finishing and finishing passes (Figure 43). The process is similar to WEDM; however, in Wire ET, the tool is not subjected to wear (therefore the electrode is not being rewound) and the surface of the workpiece is free of recast layer [126,127].

**Figure 44:**
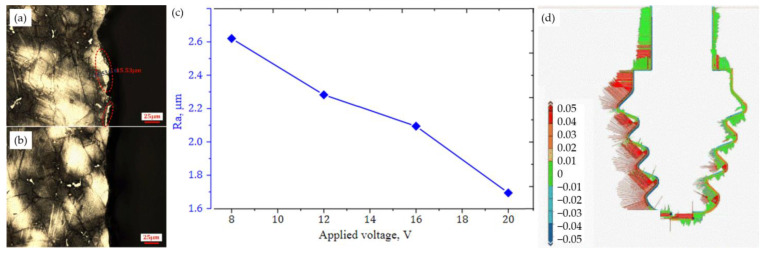
Machining results: (**a**) metallographic microsection after WEDM, (**b**) metallographic microsection after Wire ET, (**c**) surface roughness Ra, (**d**) distribution of deviations of the slot profile after WEDM (left) and after Wire ET (right) [126]. “Reprinted/adapted with permission from Ref. [121]. 2020, Elsevier”.

The main drawback of this method is the accumulation of machining products in the working gap, which can stop the process. Figure 44 presents the results of machining fir tree slots made from Inconel 718 alloy, with 20 mm height, using a 0.5 mm electrode. The depth of cut was 10 μm and the feed rate was 7.2 mm/min [126].

Wire ET allowed the recast layer to be removed after the previous WEDM. The obtained surface roughness was equal to Ra = 1.7 μm and was lower than that obtained after WEDM. Figure 44d presents the distribution of deviations of the fir tree slot profile after WEDM (left side) and after Wire ET (right side). After WEDM deviations of up to +0.04 mm were obtained, whereas after Wire ET, the deviations were in the range of −0.01 mm to +0.01 mm [126].

### 3.10. Abrasive Water Jet

**Figure 45:**
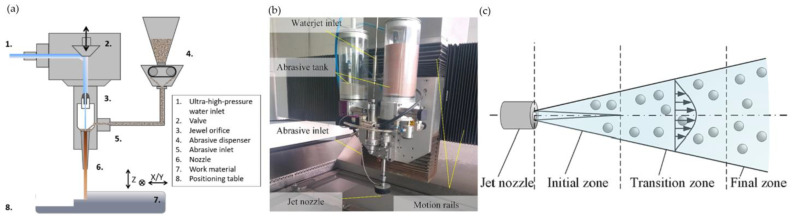
AWJM schematic diagram (**a**), water and abrasive mixing chamber (**b**), water jet zones (**c**) [128,129]. “Reprinted/adapted with permission from Ref. [129]. 2020, Elsevier”.

Among all the methods allowing to manufacture fir tree slots, one should include abrasive water jet machining (AWJM). A schematic diagram of the process and water and abrasive mixing chamber is presented in Figure 45a or Figure 45b. The removal of workpiece material in this method is possible due to a high-speed water jet mixed with grains of abrasive material (Figure 45c). The jet can be divided into an initial zone between the jet nozzle and the workpiece, a transition zone used for material erosion and a final zone where the jet leaves the workpiece [128].

**Figure 46:**
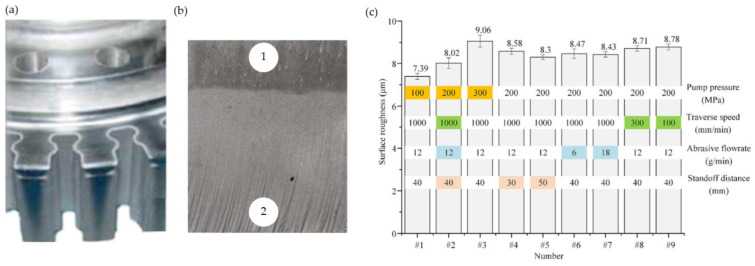
Fir tree slot after AWJM (**a**), machining marks: 1—upper area with no visible machining marks, 2—lower area with visible machining marks and striations (**b**) surface roughness Sa of Inconel 718 (**c**) [36,129,132]. “Reprinted/adapted with permission from Refs. [36,129]. 2009, 2023, Elsevier”.

AWJM results in a characteristic slant of the machined surface as well as the presence of large-sized machining marks (Figure 46b). Having no heat-affected zone is the advantage of this machining method. On the other hand, low jet coherence that can lead to a reduction in machining accuracy and the porosity of the machined surface are among the drawbacks of abrasive water jet machining [36,130,131].

In order to achieve high accuracy, several finishing passes are required in AWJ machining [128]. Figure 46a/b presents fir tree slots and surface roughness Sa of Inconel 718 alloy after abrasive water jet machining. Machining parameters are listed in Figure 46c [129].

The surface roughness and machining accuracy possible to achieve in AWJM limit the use of this method to pre-slotting only [36,130,131].

## 4. Conclusions and Outlook

Considering the requirements of the aerospace industry, environmental regulations as well as described methods of machining fir tree slots, the need to replace broaching with alternative manufacturing solutions can be observed. Currently, satisfactory results are obtained with form milling and grinding. However, these methods are associated with rapid tool wear as well as complex tool geometry, which is time-consuming to manufacture and regenerate. Promising results can be achieved with erosion machining methods. Some of them can be applied in the roughing stage—pre-slotting, due to unacceptable defects in the surface layer and HAZ (EDM, WEDM) as well as low machining accuracy (AWJM). Among the erosion methods, electrochemical machining, WECM and Wire ET allow the best results to be obtained. They can be implemented in mass production for finishing machining. There are no defects in the surface layer and the tool is subject to almost no wear. In addition, the technology itself is very flexible in terms of variations in the machined slot profile.

Table 1 presents a summary of the advantages and disadvantages of the currently used and proposed machining methods of fir tree slots in turbine discs.

The review of the literature allows us to conclude that many papers focus on research on the surface quality or the machining accuracy of fir tree slots. However, no detailed research on the economic aspects of slot machining can be found. Further research could focus on machining efficiency as well as production costs and energy consumption for each of the presented processes. Further research should also focus on the removal of defects accompanying EDM and WEDM, such as a recast layer on the machined surface, microcracks or residual tensile stresses in the surface layer. Methods that allow the introduction of compressive stresses in the surface layer should also be verified. Furthermore, searching for alternative methods of manufacturing arc fir tree slots should continue. Currently, the leading methods of manufacturing such slots are milling and grinding. ECM or EDM could be viable methods of machining arc slots. However, the disadvantages involving these methods require further research in order to optimize their performance.

## Figures and Tables

**Table 1:** Advantages and disadvantages of fir-tree-slot-manufacturing methods.

Machining Method	Broaching	Milling	Grinding	ECPG	ECM	EDM	WEDM	WECM	Wire ET	AWJM
Shape and dimensional accuracy										
Surface roughness										
Surface layer condition										
Machining efficiency										
Roughing machining										
Finishing machining										
Cost										
Tool										
Production flexibility										
Mass production										
Small lot production										

Evaluation: ▀—favorable; ▀—unfavorable; ▀—neutral.

## Data Availability

Data are contained within the article.
